# Sex and gender considerations in Alzheimer’s disease: The Women’s Brain Project contribution

**DOI:** 10.3389/fnagi.2023.1105620

**Published:** 2023-03-30

**Authors:** Laura Castro-Aldrete, Michele V. Moser, Guido Putignano, Maria Teresa Ferretti, Annemarie Schumacher Dimech, Antonella Santuccione Chadha

**Affiliations:** ^1^Women’s Brain Project, Guntershausen bei Aadorf, Switzerland; ^2^Faculty of Medicine and Health Sciences, University of Lucerne, Lucerne, Switzerland; ^3^Altoida Inc., Washington, DC, United States

**Keywords:** Alzheimer’s disease, sex differences, digital therapeutics, precision medicine, Women’s Brain Project, artificial intelligence, gender

## Abstract

The global population is expected to have about 131.5 million people living with Alzheimer’s disease (AD) and other dementias by 2050, posing a severe health crisis. Dementia is a progressive neurodegenerative condition that gradually impairs physical and cognitive functions. Dementia has a variety of causes, symptoms, and heterogeneity concerning the influence of sex on prevalence, risk factors, and outcomes. The proportion of male-to-female prevalence varies based on the type of dementia. Despite some types of dementia being more common in men, women have a greater lifetime risk of developing dementia. AD is the most common form of dementia in which approximately two-thirds of the affected persons are women. Profound sex and gender differences in physiology and pharmacokinetic and pharmacodynamic interactions have increasingly been identified. As a result, new approaches to dementia diagnosis, care, and patient journeys should be considered. In the heart of a rapidly aging worldwide population, the Women’s Brain Project (WBP) was born from the necessity to address the sex and gender gap in AD. WBP is now a well-established international non-profit organization with a global multidisciplinary team of experts studying sex and gender determinants in the brain and mental health. WBP works with different stakeholders worldwide to help change perceptions and reduce sex biases in clinical and preclinical research and policy frameworks. With its strong female leadership, WBP is an example of the importance of female professionals’ work in the field of dementia research. WBP-led peer-reviewed papers, articles, books, lectures, and various initiatives in the policy and advocacy space have profoundly impacted the community and driven global discussion. WBP is now in the initial phases of establishing the world’s first Sex and Gender Precision Medicine Institute. This review highlights the contributions of the WBP team to the field of AD. This review aims to increase awareness of potentially important aspects of basic science, clinical outcomes, digital health, policy framework and provide the research community with potential challenges and research suggestions to leverage sex and gender differences. Finally, at the end of the review, we briefly touch upon our progress and contribution toward sex and gender inclusion beyond Alzheimer’s disease.

## 1. Introduction

Alzheimer’s disease (AD) is the most common type of dementia. It is now well established that the natural history of the disease involves several stages that precede full-blown dementia. Amyloid plaques, composed of aggregated beta-amyloid (Aβ) protein, start accumulating in the brains of AD patients up to 20 years before symptoms (Fagan et al., 2014; Palmqvist et al., 2017). Initially, the brain can cope and compensate for the neurotoxicity of this process in what is called “preclinical AD.” However, often linked to the spreading of tau pathology in cortical regions, symptoms start to appear in what is called “mild cognitive impairment” or “prodromal AD.” The cognitive function progressively deteriorates, leading to dementia in a few years. When considering the AD continuum, hence also including preclinical and prodromal phases, global figures on the number of people suffering from AD were projected to be 32, 69, and 315 million in AD dementia, prodromal AD, and preclinical AD, respectively (Gustavsson et al., 2022). Considering these numbers preclinical AD stage accounts for 17% of all persons aged 50 and above and 52% of those were women (Gustavsson et al., 2022).

In fact, AD has been shown to be more frequent in women than in men (Martin Prince et al., 2015). Since aging is one of the most critical risk factors in developing AD, the higher female frequency has often been associated with men’s shorter life span than women (Kim et al., 2015; Podcasy and Epperson, 2016); however, increasing evidence indicates that this is not the only reason; biological as well as socio-cultural mechanisms are probably at play.

The biological traits that differ between men and women are referred to as sex. These are genetically defined physical features that result from the expression of sex chromosomes and are generated throughout puberty by hormonal stimulation. On the other hand, gender is a socio-cultural concept that includes behaviors attributed to being feminine and masculine that are specific to a given culture. Each society has culturally imposed behavioral and temperamental features that are deemed proper for males and females, resulting in gender norms, roles, stereotypes, and, consequently, disparities that impact aspects such as education, employment, and income.

Both sex and gender are determinants of health, according to the World Health Organization [WHO], 2021. However, when WBP was founded in 2016, the role of sex and gender was seldom acknowledged, and their study was considered a niche topic. For instance, the higher frequency among women living with AD was rarely acknowledged, and most scientists considered it negligible in clinical research. Furthermore, the sex and the gender of individuals involved in clinical development were often not described nor analyzed explicitly with regard to the disease’s characteristics. In the same way, the sex of the animals used in preclinical studies was often not reported or discussed. In terms of dementia care, the burden of caregivers (including emotional and financial), which mainly falls on the female population, was not recognized or addressed by specific policy actions at the time.

WBP was founded in Switzerland in 2017 as a non-profit association to study sex and gender determinants of the brain and mental health as the gateway to precision medicine. It is composed of professionals hailing from different disciplines with strong female leadership.

At the time of writing, WBP has contributed to more than 50 papers and 20 policy-led documents, published 6 books, given over 150 talks and lectures, and engaged in more than 50 collaborations with different stakeholders. Its work has had a profound impact on the community and has inspired several other organizations, resulting in the strengthen of *ad hoc* working groups, such as the Coordinating Panel of Diversity Equity and Inclusion at the European Academy of Neurology (EAN), the Gender mainstreaming group at OECD and the Center for Gender Medicine (CfGM) at Karolinska Institutet. Currently, WBP is now in the initial phases of establishing the world’s first Sex and Gender Precision Medicine Institute.

Since its conception, WBP has been focused on pushing the boundaries of sex and gender in AD, as our organization grew over the years, we have helped and worked with experts from other areas apart from AD. Therefore, the scientific work we have done in AD in terms of basic research, clinical applications, outreach, and policy was cross-applied by our collaborators and us to other diseases with success. Therefore, in this article we first highlight our main contributions to the field of AD in terms of basic science, clinical outcomes, digital health and policy framework. From our findings, we provide the research community with practical suggestions to leverage sex and gender differences in research studies and finally, we briefly touch upon our contribution toward sex and gender inclusion beyond AD.

## 2. The Women’s Brain Project

Over the past 5 years, WBP has contributed to the identification of profound differences in brain and mental diseases at large, and in AD in particular. The studies have revealed that such differences are complex and multifaceted, involving both biological (sex) and socio-cultural (gender) aspects; they interest all levels of research, from basic to clinical, and can also be found in novel technologies. Therefore, to properly address the complexity of the topic, the activity of the WBP has developed around four main pillars of interest: basic science, clinical science, digital biomarkers, and socio-economic determinants of health.

Biological sex differences, from genetics to hormones, can profoundly affect disease mechanisms as well as drug development. Unfortunately, the sex of the animals used in preclinical studies is often not reported or discussed. Such lack of consideration of the sex of animals in preclinical studies leads to a knowledge gap and has likely hindered therapeutic innovation. The basic science study of sex differences in brain physiology and drug mechanism of action is the first pillar of WBP’s work.

Clinical differences between men and women are crucial areas of interest in the WBP’s work. These refer to physiology, pharmacokinetics and pharmacodynamics interactions as well as symptoms (e.g., Butlen-Ducuing et al., 2021); identifying such differences calls for tailored approaches in diagnosis, care and patient journeys in AD but also in other diseases (Liberale et al., 2018).

Thanks to the advent of high-throughput advanced technologies, statistical models and computational tools, we now have novel potential digital biomarkers for early diagnosis of AD. The role of sex and gender in novel digital health technologies particularly for AD, is another pillar of WBP activities.

Finally, the study of differences between men and women cannot neglect the role of gender, meant as the socio-economic and socio-cultural construct of being a man or a woman in society. As socio-economic determinants of health are modifiable, a significant line of WBP activities has focused on policy-related projects to highlight existing gender differences and gender-based inequity in these determinants of health and how policymakers could address them.

The results gathered in the past 5 years based on these four pillars have been collected in a first textbook on the topic, “Sex and Gender Differences in Alzheimer’s Disease” (Ferretti et al., 2021). In the following sections, we highlight some of the major contributions and lessons learned by the team with useful recommendations.

## 3. Importance of sex differences in preclinical Alzheimer’s research

The presence of gonadal hormones and hormone cycles represents the first crucial biological difference between men and women, which are genetically driven. Different studies have shown the underpinning role of the sex chromosome complement, X chromosome inactivation, and environmental and epigenetic regulators in sex differences and their role in brain diseases, as we described in Pallier et al. (2022). The X chromosome transcriptome accounts for a significant fraction of the genome in both men and women. Still, it is often excluded from GWAS studies due to the complexity of its statistical analysis (Ferretti and Santuccione Chadha, 2021). For this reason, little is known about the involvement of sex chromosomes in AD and only recently have studies started to highlight its role (Davis et al., 2021). Taking AD as an example, we argue that a greater need to account for the interaction between sex and X-linked gene expression is required (Ferretti and Santuccione Chadha, 2021).

When it comes to studies in animal models, it is mistakenly believed that data from preclinical studies using female animals are complicated to analyze due to the higher variance associated with the estrous cycle; as a result, there have been comparatively few preclinical investigations done using female mice, or both male and female, in many fields of neuroscience (Beery and Zucker, 2011; Karp et al., 2017; Karp and Reavey, 2019). However, it is now known that female mice are not more variable than male mice, and both should be used.

Sex-related differences in AD have implications for developing drug targets and should therefore be carefully characterized in the context of preclinical studies and drug development. To raise awareness on this topic we have dedicated a special issue in Ferretti and Galea (2018); while more studies are looking into this, many issues still remain. We suggest that mixed-sex cohorts be the starting point for preclinical research, and data should be checked for sex differences. The next step should be mixed clinical trials if there are no obvious sex differences. In preclinical and clinical contexts, examining cohorts of each sex may be acceptable if the findings indicate a sex difference, as highlighted in Butlen-Ducuing et al. (2021). It is important to note that drug pharmacokinetics (PK) and pharmacodynamics (PD) can potentially differ among the sexes and it may be crucial in these circumstances to gather PK/PD data for both sexes already in preclinical studies and then assess whether or not to incorporate a design for potential sex differences in human dosage-finding trials to determine the ideal dose.

Finally, beyond *in vivo* studies, neuroscience is quickly embracing the use of new and complex *in vitro* models of disease mechanisms and drug response. Taking AD as a case study, we currently examine how sex differences can be accounted for *in vitro*. We argue that *in vitro* models of increasing complexity should account for a sex as an experimental variable. Therefore, we propose practical recommendations as to how to investigate sex differences (if not known) or address (if known) them (Castro-Aldrete, Einsiedler et al., in preparation).

## 4. Sex and gender differences and clinical outcomes in Alzheimer’s disease

Several differences in clinical phenotypes have been described among men and women living with AD. We have summarized such differences in several reviews (Ferretti et al., 2018, 2020; Martinkova et al., 2021), a dedicated special issue (Mielke et al., 2018), as well as in the different textbooks on the topic (Abdelnour et al., 2021; Moro et al., 2022). In sum, the body of data suggests that sex is an essential component in the phenotypic heterogeneity of AD and should not be overlooked in clinical practice or preclinical research. Therefore, the analysis and reporting of sex differences in clinical investigations must be significantly improved to create strong enough data to guide clinical practice and policy (Ferretti et al., 2018; Ferretti and Galea, 2018; Hampel et al., 2018b).

Interestingly, the available evidence indicates significant sex and gender differences along the AD continuum, which are disease-stage dependent. Women appear to be relatively protected than males during the prodromal stages; interestingly, women display better cognitive performance for the same amount of hippocampal neurodegeneration (Sundermann et al., 2016). In our own work in collaboration with Alzheimer’s Disease Precision Medicine Initiative” (APMI), we have found sex differences in AD biomarkers of amyloidosis, neurodegeneration, and rsFC in cognitively intact individuals older than 70 years. In particular, male compared with female sex have higher accumulation of *in vivo* brain amyloid load in the anterior cingulate cortex. This indicates that a greater amyloid load is necessary before men manifest symptoms (Cavedo et al., 2018). This clinical difference in prodromal phases is not unique to AD and we have contributed to its study also in other fields; for instance, in the behavioral variant of frontotemporal dementia (bvFTS), women show larger behavioral and executive reserve than men, and neurodegeneration in women must be more severe to cause symptoms comparable to those in males (Illán-Gala et al., 2021).

In contrast with the female protection observed at early stages, it has been shown that after clinical diagnosis, women with mild-cognitive impairment (MCI) progress twice as fast as men (Lin et al., 2015), a result with potential implications for the management of patients as well as for clinical trial design. A very interesting line of research indicates that women in the early stages of AD might miss diagnosis based on standard neuropsychological tests. As women outperform men in verbal memory, these scales tend to be too easy for women and miss the beginning of the pathological process; women would therefore be diagnosed at later stages, which might explain the faster decline observed after diagnosis, as women are more advanced in the disease trajectory (Sundermann et al., 2021). This sex and gender difference is only one, but a powerful example of patient heterogeneity.

To address patient heterogeneity, the field of medicine is moving in the direction of biomarker-based precision medicine. While well advanced in other fields such as oncology, addressing patient heterogenicity is still in its infancy in neurology. In AD, several biomarkers, including imaging, fluid and digital ones, have been developed and are starting to be employed more and more in research and the clinical context in recent years. The diagnostic and prognostic value of such biomarkers can differ for men and women, including in preclinical stages (WBP, manuscript in preparation). Together with the APMI and cohort program (APMI-CP), the WBP is advocating for a new, AI-powered, biomarker-based clinical framework, which should be implemented to close the sex gap (Hampel et al., 2018a). However, a sex-sensitive clinical diagnosis of AD dementia based on biomarkers is still needed.

Alzheimer’s disease risk factors can be different for men and women. Evidence for sex-specific vulnerability to APOE4 is growing (Farrer et al., 1997; Altmann et al., 2014; Neu et al., 2017), and the effects of sex-genotype interactions on responses to hormone replacement therapy and cholinesterase inhibitors have been observed (Macgowan et al., 1998; Holland et al., 2013; Jack et al., 2015). Midlife cardiovascular risk factors, for instance, are linked to a higher risk of developing dementia in women than men (Huo et al., 2022).

Although the WBP has emphasized largely on APOE4 studies as a risk factor with sex and gender considerations in AD, we would like to highlight that due to the multifactorial and heterogeneous nature that characterizes AD, apart from APOE4, there might be other risk factors that can affect females or males risk of developing AD. Such genetic and modifiable risk factors have been described elsewhere in excellent detail (Baumgart et al., 2015; Bellenguez et al., 2022).

In addition to genetic risk factors, modifiable, life-style related risk factors are well known to affect risk (as much as 40%) of AD (Livingston et al., 2020). It is important to highlight that most of such modifiable risk factors are known to occur differently across sexes and genders since inequities are not only linked to biological sex but also to gender, as summarized by Ferretti et al. (2020).

According to Zhang et al. (2021), factors such as psychological state, level of engagement in society, pre-existing conditions such as diabetes and traumatic brain injury (TBI), and lifestyle habits can change a person’s risk levels to develop AD. For example, different studies have demonstrated the risk between diabetes and AD (Winkler et al., 2014; Espeland et al., 2018; Marseglia et al., 2018). Furthermore, a positive correlation between TBI and AD has been described in a Danish study that explored different levels of injury and their impact on AD (Nordström and Nordström, 2018).

Regarding modifiable vascular and lifestyle-related risk factors, the Finnish Geriatric Intervention Study to Prevent Cognitive Impairment and Disability (FINGER) has demonstrated that a multidomain intervention could improve or maintain cognitive functioning in at-risk elderly people from the general population (Ngandu et al., 2015).

The occurrence of sex and gender differences in risk factors calls for tailored preventative campaigns for men and women and might also be a key element to consider in patient stratification for clinical trials and overall study design.

The emergence of highly diverse subgroups of patients with distinct risk factors, comorbidities, illness trajectories and likely specific neurobiology would require a new approach where the generated data can be multiplexed in a combinatorial approach with clinical, biomarker and other omic data to produce algorithms for the prediction, diagnosis, prognosis and treatment of AD, analogous to the situation in oncology (Ferretti and Santuccione Chadha, 2021). The lack of consideration of such heterogeneity has severely hindered our ability to identify at-risk individuals early and discover successful treatments in clinical trials. Therefore, if criteria for patient admittance into trials are created, sex may be taken into account alongside other variables (genetic, biomarker status) to generate a biologically homogeneous sample (Ferretti et al., 2020).

In our meta-analysis of 56 randomized clinical trials for AD, we have shown that only 12.5% of articles reported data stratified by sex (Martinkova et al., 2021); therefore, we advocate for more transparent reporting of results, even when negative. In the same study, we determined that of the 39 575 total participants with AD included in the clinical trials examined, women represented 59.0% of patients, a number that lowered to 57.9% of women when we examined only experimental drugs that had not been approved. As women represent up to 65% of the real-world population living with AD, these data indicated that female patients might have specific barriers to accessing clinical trials. Some might be due to specific criteria that systematically exclude women, such as lower educational level (Rosende-Roca et al., 2021), however, additional barriers might exist and need to be identified across the patient journey, as discussed in the next sections.

## 5. Inclusion of sex and gender in digital health applications for Alzheimer’s disease

As in all areas of our lives, novel technologies are emerging also in AD and will most likely become crucial tools to support the patient journey in AD.

In fact, technological innovations in digital technologies and data analytics provide an umbrella of opportunities to estimate health variables to improve personalized health outcomes. Digital Health refers to the use of data and communication technologies to promote wellness and, in some instances, manage illnesses. Digital health technologies use computing platforms, software and sensors that span many uses. Examples of Digital Health technologies are mobile medical apps intended to improve clinical decisions to diagnose and treat diseases. These apps collect users’ data which are subsequently stored and analyzed to enhance health status. Digital biomarkers and predictive algorithms promise to dramatically change the landscape of medicine, greatly improving and streamlining health management, from risk factors monitoring to diagnosis and treatment.

The field is developing today the technologies that will be used in the next decades and it is important to be aware of sex and gender aspects also in this field. Bias is a hidden issue of most databases used to generate algorithms; as such the risk, as highlighted in other fields using artificial intelligence (AI), is to generate biased tools that do not serve the whole population. The AD community needs to investigate whether gender biases might affect the efficiency of AI tools for health. At the same time, sex-specific characteristics might be leveraged to improve the efficiency of such digital tools. For this reason, the WBP has a dedicated working group focusing on this topic and has contributed to our understanding of how even such technologies must consider sex and gender aspects.

The popularization of digital biomarkers could change the course of clinical research, especially concerning AD. In a first article, we gathered experts in novel technologies and identified sex as a factor that should be included in all digital biomarker algorithms (Cirillo et al., 2020). This is particularly relevant also in AD risk assessment, diagnosis, and clinical treatment and promises to allow more personalized and accurate care leading to better disease management (Ferretti et al., 2020; Sundermann et al., 2021).

Most studies in the past have used pooled data (male and female) to generate predictive algorithms for risk prediction based on genetic or phenotypic and clinical patient data. Importantly, recent studies have shown that sex is a key aspect of such algorithms (van Maurik et al., 2019); in some cases, one can even increase the predictive value by creating stratified algorithms by sex (Fan et al., 2020). There is, therefore, a strong case for a careful analysis of sex differences in datasets, as these can be leveraged to make digital tools more powerful. This is what we called in our paper a “desirable bias” (Cirillo et al., 2020).

On the other hand, until last year, it was not known whether sex differences would be also present also in digital biomarkers. To study this, the WBP has partnered with Altoida Inc., which has created the Neuro-Motor Index (NMI), a digital biomarker application dedicated to early AD diagnosis. The NMI measures cognition *via* augmented reality (AR) and motor skills in the fingers with an aim to replicate daily activities and tasks (Buegler et al., 2020).

The WBP-led study analyzed data from clinical settings (*N* = 438 patients from Italy, Greece, and Spain) and data from the Japanese population (*N* = 130 patients) (Harms et al., 2022). In this study, the clinical data consists of controlled tests of elderly subjects with MCI, AD or Aβ (+) biomarkers or young healthy controls. Each patient completed a series of tests on hand-held electronic devices supplied with the Altoida Inc., application to produce data points. We used all of this data (which was devoid of anagraphical information) to train an algorithm to determine the sex of the patient based on the data received. The sex classifier successfully determined the sex of the patient among healthy patients, but its power faded in MCI and AD populations (Harms et al., 2022). Notably, the successful differentiation in healthy individuals shows that males and females have distinct differences in their neurocognition as detected by the NMI. These results prove that the sex of a patient can affect digital biomarkers; more studies will be needed to confirm and explain these findings. Based on these results, we advocate for digital biomarker programs to factor in sex when gathering data, in this case, for AD diagnosis and treatment design.

Digital biomarkers are only one example of the power of AI-driven solutions in healthcare. AI-powered data analysis can detect specific patterns that can be leveraged to improve therapeutic and preventative measures against diseases at large. While this field it’s just at its inception in AD, it is much more developed in other branches of medicine.

For example, the value of AI in medicine has been highlighted during the Coronavirus Disease 2019 (COVID-19) pandemic, which has revealed how unprepared healthcare systems are. At the WBP, we believe that AI is the key to preparing the healthcare system for future pandemics and, in general, addressing unmet medical needs such as AD. AI aims to incorporate patient data to make informed decisions concerning patient care and treatment plans.

Integrated AI could improve, among other categories, the triage, diagnosis and risk prevention in the scenario of a pandemic (Santus et al., 2021). A major benefit of AI is its ability to examine large volumes of information being published in various scientific journals and determine what is “good” and what is “bad” according to a previously established set of criteria within a short time frame. AI can also help drug repurposing by developing algorithms that identify and analyze protein-protein interactions. Recently, using AI, baricitinib was identified and has shown success in fighting against COVID-19 (Richardson et al., 2020).

The WBP supports the idea that by using desirable and undesirable bias exclusion, AI can accurately help reduce unnecessary sex and discrimination among genders (Cirillo et al., 2020; Santus et al., 2021). Using desirable bias AI can help in more precise and effective diagnostics for females and male (Stanovsky et al., 2020; Castaneda et al., 2022). For example, the training of AI algorithms can potentially increase accuracy if sex is considered (Straw and Wu, 2022).

## 6. Sex and gender policy framework in Alzheimer’s disease

Constant legislative and policy adaptation to current technological innovation in a rapidly evolving field such as AD is vital for trustworthy relationships. Policy includes many facets of healthcare, and policy framework in clinical trials is only one of them. At the core of WBP we are working toward bridging science and society, educating policymakers and raising awareness on important societal and economic aspects linked to brain health, including sex and gender differences.

The work of the WBP in policy and advocacy includes response to the global policy agenda by contributing to ongoing initiatives as well as driving WBP-led projects and deliverables with evidence generation.

Indeed, gender (meant here as the socio-cultural construct of being a man or a woman in the society) influences a number of crucial health determinants, including wealth, education, access to healthcare and behaviors. An example of a gendered expectation that profoundly affects health in AD is the caregiving burden, which is currently overwhelmingly carried by women worldwide. In papers, books as well as policy reports (Erol et al., 2015; Alzheimer’s Disease International and McGill University, 2022) we have highlighted the role of women as caregivers and the need for *ad hoc*, tailored support measures for men and women as caregivers, based on their specific needs.

Specifically, in AD, we have co-authored several policy reports on the role of sex and gender, including sexuality (Gove et al., 2022). In an invited chapter of the World Alzheimer’s report (Alzheimer’s Disease International and McGill University, 2022) we have described how sex and gender differences can influence the diagnostic pathway. In particular, gender can affect the speed of diagnosis. Our research and the cumulative evidence in the past years indicate that sex and gender can affect the patient journey even beyond diagnosis, beginning with arranging an appointment for a routine checkup and ending with receiving treatment for an illness or injury. To further our understanding of sex and gender impact on AD patients, in a recent WBP-led survey-based study, we have found key differences in symptom detection, support attitude, treatment regimens, and disease management in the AD patient journey in men and women (Quevenco et al., 2023). This is important for policymakers as tailoring patient journeys for each sex can help the healthcare systems at several levels, from reducing the gap in disease awareness and health-seeking behavior, developing strong preventative routines, encouraging early diagnosis and allowing patients to stay as healthy as possible for as long as feasible. WBP has also contributed to the WHO blueprint for dementia research by highlighting the importance of incorporating sex and gender dimension at all stages of research (World Health Organization [WHO], 2022a).

Alzheimer’s disease is one of the most prevalent and costly disease for society and many WBP-led initiatives for evidence generation are geared to address this issue. WBP members have acted as expert advisors in a study detailing neurological disorders’ economic burden (Economist Impact, 2022). However, to drive future policy changes, it is important to understand also the economic dimension of gender medicine in neurology. To do so, the WBP has commissioned a health economic study focusing on 5 main disorders (AD, PD, MS, stroke and migraine) to identify major sex and gender differences that can affect healthcare costs and how their consideration could lead to more cost-effective health systems (Economist Impact, 2023).

Given the socio-economic costs of psychiatric and neurological disorders, it is important to characterize further and understand these differences in the larger context of brain health. To do this, we have contributed to the study describing the results of the largest survey to date on perceptions of brain health. This study documented that, in general, the lay public has a low level of awareness of brain health and its risks; differences in awareness of diseases in general and risk among men and women were observed. For example, men are less likely than women to consider factors such as substance use, sleeping habits and diet as having an influence on the brain health (Budin-Ljøsne et al., 2022). These results are important for driving *ad hoc* communication campaigns to educate and raise awareness of brain health in the population, which is key for disease prevention in society.

The importance of brain health in the global agenda has been underscored recently by the, WHO position paper on brain health, to which WBP has contributed (World Health Organization [WHO], 2022c). A particularly important topic, also relevant for AD, is the intersection between brain health and aging. Prevention of age-related disorders is increasingly becoming a key global goal, and to support the discussion in this field WBP is working alongside other partners to understand longevity in the context of brain health.

Together with the OECD the WBP is demonstrating the importance of building and protecting our own brain health, which we call “late-life Brain Capital.” We have argued that investing in late-life Brain Capital can help older persons retain, engage, and empower themselves (Dawson et al., 2022). A combination of public health strategies targeting tobacco use, blood pressure control, cardiovascular disease management and prevention, among others, can reduce the likelihood of cognitive decline by making an investment in late-life brain capital. To be effective, these actions need to consider specific needs of segments of the population, including sex and gender differences.

The study of sex and gender differences is highly relevant for policymakers as it can support the development of strategies for prevention, early detection and better treatment of diseases that present a huge medical need and socio-economic cost. However, we realize that sex and gender-sensitive medicine is still in its infancy in neurology and psychiatry. The WBP has therefore convened a dedicated series of regulatory roundtables, now at its third edition, to discuss the best way to integrate sex and gender-sensitive medicine in developing solutions for neurological patients.

To obtain a comprehensive picture, in collaboration with the Task Force on gender and diversity of the EAN, we have mapped current European research, financing, and teaching initiatives that incorporate sex and gender considerations in neuroscience and neurology (Hentzen et al., 2022). We demonstrated that there is a rising demand and interest in neurological domains, both from funding organizations and researchers. However, most activities, particularly in education, are linked to individual researcher motivation and are rarely organically embedded into the curriculum and strategic research goals (Hentzen et al., 2022).

For this reason, the WBP is currently in the process of establishing a foundation and a dedicated research institute to strive for innovation in sex and gender-sensitive precision medicine for brain and mental disorders.

## 7. Future challenges and recommendations

**FIGURE 1:**
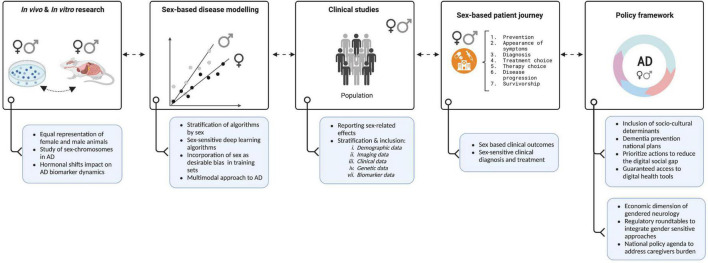
The Women’s Brain Project toward a sex-sensitive Alzheimer’s disease (AD) approach. Figure shows key recommendations (blue boxes) from which Alzheimer’s and brain research milestones (white boxes) will benefit in constant feedback to accurately capture the impact of sex and gender and develop precision medicine agendas. Created with Biorender.com.

Although researchers have become increasingly aware of the need to consider the impact of sex and gender on the development and progression of AD, the growing body of research in this area would benefit from carefully addressing current and potential future challenges. In this regard, we have graphically summarized in Figure 1 recommendations from which AD research will benefit to accurately capture the impact of sex and gender. Furthermore, in Table 1 we provide key resources that can be a starting point in conducting sex and gender research in AD.

**TABLE 1:** Useful resources for conducting sex and gender research.

Type of resource	Name	Key aspect	References
Courses	Bench to bedside: integrating sex and gender to improve human health course	Free online course by the US National Institutes of Health. It is divided into 6 thematic modules: immunology cardiovascular disease pulmonary disease neurology endocrinology mental health	National Institutes of Health, 2023
	Sex as a biological variable (SABV): a primer	Free online course by the US National Institutes of Health.	Dance, 2019
	Canadian Institutes of Health Research	Includes courses about sex and gender in health research	CIHR, 2019
	Statistical considerations for sex inclusion in basic science research	A recorded presentation explaining statistical and sample-size considerations for including sex as a biological variable.	BlueJeans, 2016
Online tool	Gendered innovations	This tool helps in developing methods to do analysis on the basis of sex and gender, how sex and gender analysis helpful in innovations	European Union, 2011
	Sex and gender research methods	Canadian Institutes of Health Research, is a series of methods articles aimed at equiping researchers with practical tips and tools from prominent researchers on integrating sex, gender, and other identity factors into various fields of health research.	Canadian Institutes of Health Research, 2022
	Genderedinnovations.se	It is developed at Karolinska Institute, is the Swedish version of Stanford’s Gendered Innovations and contains useful content in the form of Swedish expertise, experience, tools, videos, and case studies.	Gendered Innovations, 2019
Database	Janusmed sex and gender	It has information related to sex and gender aspects for drug treatment	Janusinfo, 2022
	Drug trial snapshot	It provide information to consumers and healthcare professionals about participation in clinical trials	Drug Trials Snapshots, 2022
	GenderMed database	It provide literature research that addresses sex and gender differences	Oertelt-Prigione et al., 2014
	Gender experts	Database related to women experts on gender equality.	Gender Experts, 2021
Organizations	Gender equality academy	Horizon 2020 project developing and implementing a high-quality capacity-building programme on gender equality in research, innovation, and higher education.	Ge-academy, 2019
	LIBRA	EC-funded project that brings together ten research institutes in ten European countries to promote gender equality in life sciences.	LIBRA, 2021
	PORTIA	UK not-for-profit organization with extensive expertise in EU gender and STEM policy.	PORTIA, 2020
	NIH ORWH	US National Institutes of Health’s office for research on women’s health	Research on Women’s Health, 2023
	Institute of gender in medicine	Focuses on interventions to promote healthy behaviors and how such interventions can be designed in a gender and diversity sensitive way.	Institute of Gender in Medicine, 2023
Recommendations	The promises and pitfalls of sex difference research	Discuss issues related to inclusion of both sexes to specialization of sex differences with attention paid to statistics and the need for sex-specific treatments.	Galea et al., 2020
	Sex and gender differences research design for basic, clinical and population studies: essentials for investigators	Compilation of sex and gender studies to excrete basic causes of diseases and avoid a reflexive attribution of seeming sex differences solely to biology.	Rich-Edwards et al., 2018
	Biomedical research falls short at factoring in sex and gender	Equity, accuracy, and transparency in both the conduct and reporting of research in subjects of both sexes.	Shansky and Anne, 2021
	The impact of sex and gender on the multidisciplinary management of care for persons with Parkinson’s disease	Potential impact of sex and gender on care for people with PD, and identify key knowledge gaps that hamper immediate implementation of sex- or gender-sensitive approaches.	Göttgens et al., 2020
	The sex and gender dimensions of COVID-19: a narrative review of the potential underlying factors	Sex is a significant risk factor for severe disease and mortality due to coronavirus disease 2019 (COVID-19).	Taslem Mourosi et al., 2022
Books	Sex and gender differences in neurological disease	Each chapter includes the latest information on sex and gender differences in neurological disease	Moro et al., 2022
	Sex and gender bias in artificial intelligence and healthcare	Highlights the relevance of sex and gender differences and bias in the development of novel technologies for health.	Cirillo et al., 2022
	Sex and gender differences in Alzheimer’s disease	The first academic book on sex and gender differences in Alzheimer’s disease.	Abdelnour et al., 2021

Hormonal differences between men and women can play a significant role in the development and progression of AD (Mosconi et al., 2018; Rahman et al., 2019, 2020). Therefore, understanding how these differences contribute to the disease and how they can be used to develop gender-specific treatments is essential but at the moment we lack proper data. As a future direction, we need to capture hormonal related data in all clinical studies. Equally important, commonly used biomarkers, such as those found in brain scans, blood tests, and spinal fluid used to provide valuable information about the progression of AD, should evaluate whether they may differ between men and women and how hormonal shifts can impact AD biomarker-dynamics to develop gender-specific treatments (Herman et al., 2022).

Incorporating sex and gender dimensions to training sets of deep learning algorithms should become a priority. If a deep learning model is trained on a dataset that is not representative of the population, it may not accurately determine the effect of sex and gender. To address this issue, it is important that training sets account for a balanced representation of sex and gender in the desired target population. This is especially important in deep learning platforms that simulate the structure of “orphan” proteins, which are proteins for which the structure is unknown. This is important because many disease-related proteins fall into this category, including AD (Varadi et al., 2022). To the best of our knowledge, sex and gender are not disclosed nor included in the algorithm’s training set (e.g., AlphaFold). The algorithm is trained using a huge dataset of known protein structures and their matching amino acid sequences, however, this dataset does not include information about the sex of the species from which the proteins were acquired (Senior et al., 2020; Jumper et al., 2021; Varadi et al., 2022). When examining the results of these algorithms, it is critical to include sex and gender, especially when researching disease-related proteins that are known to be impacted by sex and gender. Furthermore, these algorithms may be used to discover prospective therapeutic targets that might be specific to one sex or to investigate sex-based variations in protein structure and function in the human genome (Tunyasuvunakool et al., 2021).

We envision that due to the high level of complexity, exploring the impact of social and cultural factors and its sex-related outcomes on a certain population will be one of the main challenges in AD research. Social and cultural factors are difficult to study due to the lack of standardization in their quantification making it difficult to compare results or perform a meta-analysis. Therefore, the challenges in controlling for confounding variables can influence the accuracy in determining the impact of sex and gender in AD. This is starting to be addressed by developing a multi-feature multimodal approach to neurodegeneration that includes among other variables, sociodemographic information (Moguilner et al., 2022).

With the progression of AD, patients became heavily dependent on their caregivers for everyday functions, which have significant implications not only for them but also for their caregivers. The correlation between low education levels and the burden of caregiving appears to affect mainly women and has been reviewed elsewhere in excellent detail (Mielke, 2018). This burden on caregivers has been associated with the development of AD and other neuropsychiatric disorders, as highlighted in different studies (Shoukr et al., 2022; Hellis and Mukaetova-Ladinska, 2023). According to Figueroa et al. (2021) digital health disadvantages women, particularly those from racial or ethnic minority backgrounds, due to limited access and exclusion from app creation, gender imbalance in digital health leadership, and detrimental gender stereotypes. We believe that societies can benefit from digital health and AI approaches by developing national dementia frameworks that prioritize actions to reduce the digital gap between digitally disadvantaged and advantaged individuals. Guaranteed access to early and accurate diagnosis -including digital health-, medications, and equity in the actions needed to diminish caregiver psychological and financial burden must have high priority.

## 8. Sex and gender differences beyond Alzheimer’s disease

The WBP team has made important contributions to the study of sex and gender differences in AD, as well as its awareness in society and its consideration at the policy level. Sex and gender differences occur and are important in neurology and psychiatry well beyond AD and this is captured by the work of our group in other fields of medicine.

To give a few examples, WBP has contributed to the characterization of sex and gender aspects in traumatic brain injury (Rauen et al., 2021), neuropathic pain Parkinson’s disease and schizophrenia in a dedicated, WBP-led special issue in the journal Frontiers in Neuroendocrinology (Szoeke et al., 2020), stroke (Sandset and Ferretti, 2021) and in brain health (de Lange et al., 2021).

As an example of WBP response to the ongoing policy actions, we have contributed, as members of the OneNeurology group, to the Global Action Plan on Epilepsy and other neurological disorders by the WHO, to make sure that sex and gender aspects in neurology are part of the research agenda (World Health Organization [WHO], 2022b).

The specific needs of female migraine patients have been highlighted in several *ad hoc* policy and awareness projects, including communications campaigns, such as #notallinherhead social media campaign, directed at the lay public. Awareness campaigns and communication programs are run by the WBP team also for psychiatric disorders such as depression, anxiety and ADHD.

Interestingly, the COVID-19 pandemic has revealed the importance of sex and gender differences and the WBP has been particularly active in documenting such differences and advocating for their consideration in clinical trials (Grisold et al., 2021; Jensen et al., 2021, 2022).

Finally, we have advocated for the proper consideration of sex and gender in the context of drug development (Ferretti and Galea, 2018), particularly for neurological and psychiatric disorders that present an unmet medical need (Butlen-Ducuing et al., 2021).

## 9. Conclusion

The work done by the team at WBP showcases the power of female leadership, a diverse and multidisciplinary team in the field of Alzheimer’s research and beyond. Collaboratively the WBP have helped to change perceptions, increase visibility and reduce sex biases in preclinical research, clinical science and policy framework. Incorporating specific individual needs (including those driven by sex and gender aspects) will be key to reaching a precision medicine approach in AD, as well as personalized patient management for a more sustainable healthcare.

## Author contributions

LC-A conceived the manuscript. LC-A, MM, and MTF wrote the manuscript. LC-A, MM, and GP collected and cataloged the literature. LC-A, MTF, and ASD contributed substantially to the content discussion and reviewed/edited the manuscript before submission. ASC, MTF, and ASD provided the resources and supervision. All authors contributed to the article and approved the submitted version.
